# Characterization of auditory sensation in *C. elegans*

**DOI:** 10.52601/bpr.2024.240027

**Published:** 2024-12-31

**Authors:** Can Wang, Elizabeth A. Ronan, Adam J. Iliff, Rawan Al-Ebidi, Panagiota Kitsopoulos, Karl Grosh, Jianfeng Liu, X.Z. Shawn Xu

**Affiliations:** 1 Life Sciences Institute, University of Michigan, Ann Arbor 48109, USA; 2 Department of Molecular and Integrative Physiology, University of Michigan, Ann Arbor 48109, USA; 3 Department of Mechanical Engineering, University of Michigan, Ann Arbor 48109, USA; 4 Department of Biomedical Engineering, University of Michigan, Ann Arbor 48109, USA; 5 College of Life Science and Technology, Key Laboratory of Molecular Biophysics of MOE, Huazhong University of Science and Technology, Wuhan 430074, China

**Keywords:** *C. elegans*, Hearing, Audition, Mechanosensation, Mechanosensory

## Abstract

Research using the model organism nematode *C. elegans* has greatly facilitated our understanding of sensory biology, including touch, olfaction, taste, vision and proprioception. While hearing had long been considered to be restricted to vertebrates and some arthropods, we recently discovered that *C. elegans* is capable of sensing and responding to airborne sound in a frequency and sound source-size-dependent manner. *C. elegans* auditory sensation occurs when airborne sound physically vibrates their external cuticle (skin) to activate the sound-sensitive mechanosensory FLP/PVD neurons via nicotinic acetylcholine receptors (nAChRs), triggering aversive phonotaxis behavior. Here, we report stepwise methods to characterize these three features of *C. elegans* auditory sensation, including sound-evoked skin vibration, neuronal activation, and behavior. This approach provides an accessible platform to investigate the cellular and molecular mechanisms underlying auditory sensation and mechanotransduction mechanisms in *C. elegans*.

## INTRODUCTION

The sensory nervous system enables animals to detect and process external cues to initiate behaviors and navigate their environment (Luo 2020). There are six commonly shared sensory modalities: vision, touch, olfaction, taste, hearing, and proprioception (Luo 2020). Although individual species have evolved diverse structures specialized in detecting various sensory stimuli, they share common organizing principles (Kandel *et al*. 2012; Luo 2020). This includes the expression of molecular sensors in specialized sensory cells, which detect and transduce sensory inputs into electric and/or chemical outputs (Kandel *et al*. 2012; Luo 2020).

Despite its simple cylindrical body plan, the nematode *C. elegans* has been widely used as a genetic model for studying sensory biology (Bargmann 2006; Goodman and Sengupta 2019; Iliff and Xu 2020). By developing a custom sound delivery device that administered precise and localized sound to *C. elegans,* we discovered for the first time that auditory sensation exists in nematodes (Iliff *et al*. 2021). Remarkably, *C. elegans* elicits avoidance behavior (termed phonotaxis) in response to airborne sound in the frequency range of 100 Hz to 5 kHz when aimed at the head or tail (Iliff *et al*. 2021). We further showed that airborne sound physically vibrates the worm’s skin (cuticle), resulting in the activation of the mechanosensory neurons FLP/PVD to drive phonotaxis behavior (Iliff *et al*. 2021). Furthermore, we found that the nicotinic acetylcholine receptor (nAChR) DES-2/DEG-3 is required for auditory transduction independently of ACh, revealing an unexpected role of nAChRs in mechanosensation (Iliff *et al*. 2021). Moreover, we reported that worms optimally respond to sounds emitted from small speakers that generate sharp sound pressure gradients along the worm’s body length, indicating that worms sense sound pressure gradients rather than absolute sound pressure level (Wang *et al*. 2023).

Here, we outline the parameters required to build a custom sound-delivery system and the methods used to assay the three essential features of auditory sensation in *C. elegans* (skin vibration, neuronal activation, and sound-evoked behavior). These methods provide a robust platform to decipher the mechanisms underlying auditory sensation and mechanotransduction using the model organism *C. elegans*.

## METHODS

### Sound delivery system setup

**Figure 1:**
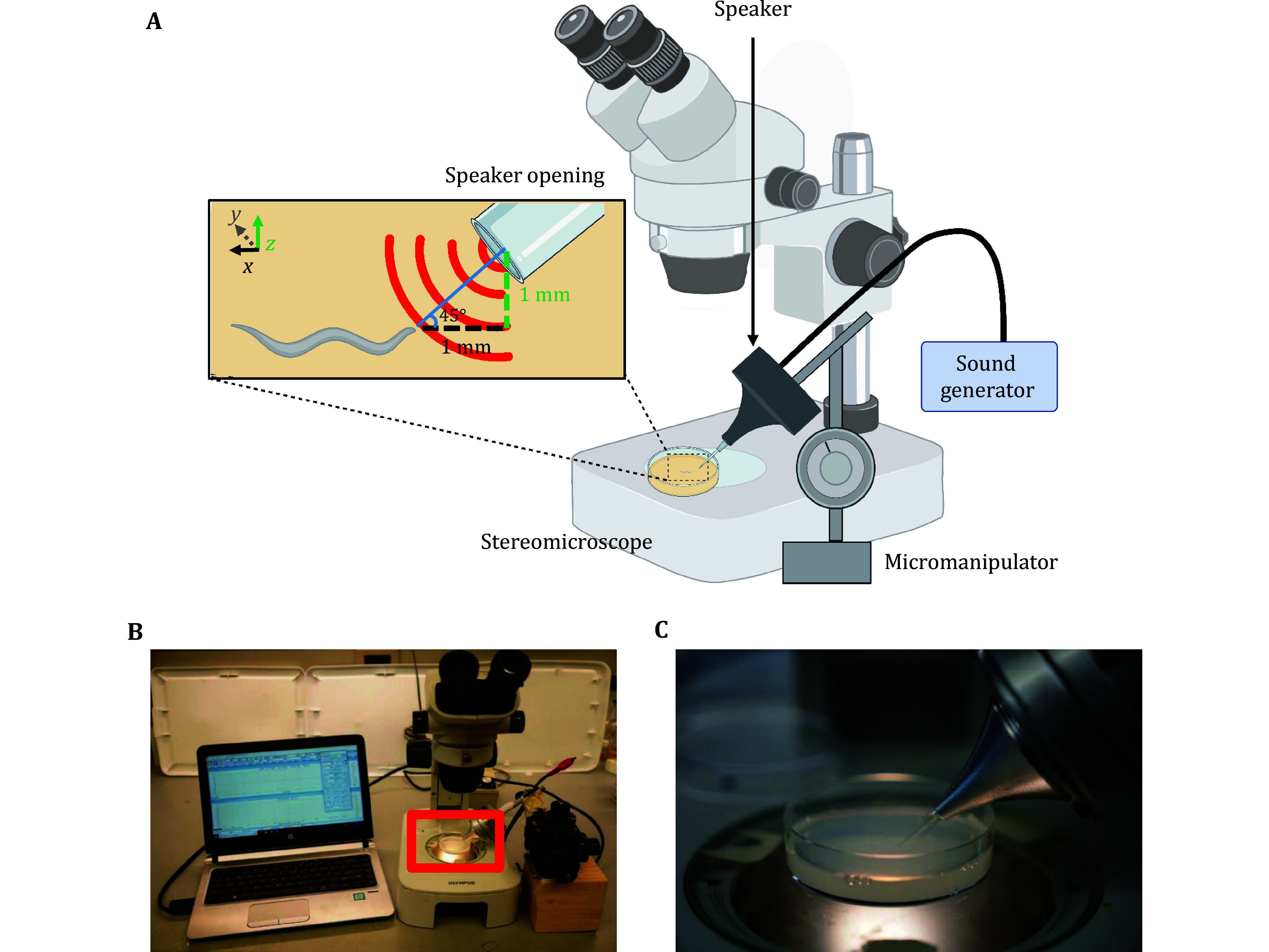
Overview of the sound delivery system for assaying *C. elegans* auditory responses. **A** Schematic describing the custom sound delivery system used to examine worm auditory responses. The sound generator (here, computer) is connected to a speaker mounted on a micromanipulator. This enables precise adjustments of the speaker opening towards the target. The speaker output port is fitted with a short length of 1/8” PVC tubing to restrict the speaker opening size, which can be further modified via the attachment of a shortened p2 pipette tip (trimmed to the desired opening size) to the open end of the tubing. The system is designed for use with a stereomicroscope to monitor worm behavior. The inset depicts the recommended distance and angle for positioning the speaker opening towards the worm. **B** Snapshot image showing the sound delivering system positioned above the stereomicroscope field of view. The micromanipulator base is mounted on a wooden block to facilitate positioning the speaker at the base of the stereomicroscope field of view. **C** Inset from Panel B, depicting a zoomed-in snapshot image of the speaker tip positioned above the NGM testing plate. The speaker tip is held 1 mm above the NGM surface at a 45° angle

In this section, we describe the design of a simple, custom sound delivery system that can be used to precisely assay *C. elegans* auditory responses (Fig. 1). We previously showed that *C. elegans* avoids localized sounds in frequencies ranging from 100 Hz to 5 kHz with lower frequencies requiring lower sound intensities (Iliff *et al*. 2021). Further characterizations of *C. elegans* auditory sensation primarily used 1 kHz sound frequency at 80 dB SPL (Iliff *et al*. 2021). We recommend replicating our sound delivery setup to evoke optimal auditory responses when first initiating these studies.

We used a computer equipped with a sound card and commercially available Multi-Instrument (MI) audio software (Virtins Technology) to generate sinusoidal tones. An RCA cable transmitted computer-generated signals to a multi-field magnetic speaker equipped with an internal parabolic cone (Tucker-Davis Technologies MF-1). We restricted the speaker’s opening by attaching a short length of 1/8” PVC tubing (inner diameter of 1.5 mm) to the speaker (Fig. 1). For behavior and vibration experiments, in addition to the PVC tubing, we attached a shortened pipette tip cut to an inner diameter opening of 0.5 mm. Based on the observation that worms specifically respond to localized sounds generated from small speakers (<3 mm inner diameter opening) (Wang *et al*. 2023), the final speaker opening size should be restricted to <3 mm to optimize auditory responses.

We mounted the speaker onto a micromanipulator arm (Narishige NMN-21) via an 8–32 threaded rod attached to the back of the speaker (Figs. 1A and 1B). For optimal sound exposure, we recommend adjusting the angle of the speaker aperture tip to 45 degrees relative to the microscope stage and securing its position (Fig. 1A). Due to the speaker’s weight, the angle may deform. This can be counteracted by reinforcement with adhesive tape. To ensure the stability of the entire setup, we mounted the micromanipulator and speaker onto a block to appropriately elevate them to the level of the microscope stage (Fig. 1B). Example settings for delivery of 1 kHz sound using this configuration with the MI software are shown in supplementary Fig. S1.

### Alternative sound delivery system components

We have explored several alternative software and hardware options that may be considered while establishing a sound delivery setup. The Data AcQuisition and Real-Time Analysis (Daqarta) software provides an affordable alternative to generate pure tones from 100 Hz to 10 kHz using a Windows computer. To increase the maximal sound intensity generated from the computer through Daqarta, we recommend coupling this with an external audio amplifier such as Zampv.3 (Parasound). Using this approach, we found that pure tones could be amplified up to 110 dB SPL maximally.

Alternatively, commercially available software and hardware can also be used to set up a precise sound delivery system. While these are typically less cost-efficient, they provide the convenience of simple assembly and ensure precise generation of pure tones with minimal background noise. We recommend AudioPrecision’s APx500 v6.0 Audio Measurement Software in tandem with their Acoustic audio analyzer/amplifier APx517B, which we have used to administer pure sound tones with a frequency range of 100 Hz to 20 kHz at intensities from 50 to 110 dB SPL.

### Sound calibration system setup

**Figure 2:**
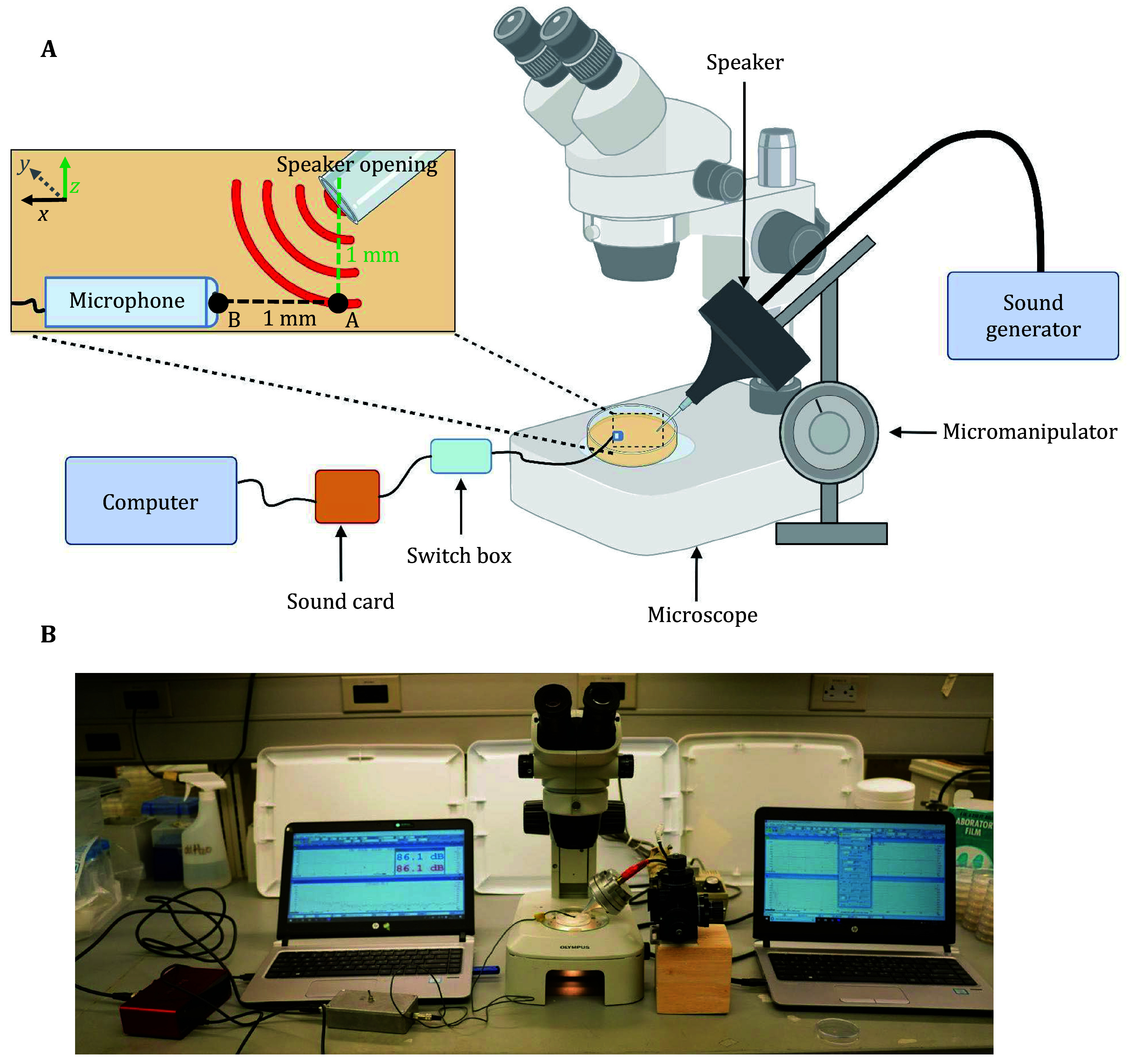
Overview of the sound calibration system setup. **A** Schematic of the sound calibration system used to ensure the emission of precise tones from the sound delivery system. The sound calibration system is composed of a computer, external sound card, switch box and mini-microphone. Calibration is performed while mimicking experimental conditions, with the mini-microphone positioned in lieu of a worm on the NGM testing plate surface. Sound intensity is calibrated to determine the input settings resulting in the desired decibel level reaching the target. **B** Snapshot image of the sound calibration system (left) and sound delivery system (right) positioned above the stereomicroscope. The mini-microphone can be secured using modeling clay

To maintain the accuracy of sound cues emitted from the sound delivery system described above, a sound calibration system is also necessary. Here we describe our calibration method that enables the delivery of sound with specific frequencies and intensities to elicit responses in the worm (Fig. 2). For calibration, we utilized the MI (Multi-Instrument) software, the same software employed for sound delivery. Alongside the MI audio software, we connected an external sound card (Focusrite Scarlett Solo 2x2 USB Audio Interface) to a custom small-diameter analog electret condenser omnidirectional microphone (1-mm inner diameter; Knowles, FG-23329-P07) to assess the acoustic properties of the generated sound fields emanating from the output port (Fig. 2). The custom miniature microphone (mini-microphone) receives power from a custom power supply and links to an external sound card through a BNC to ¼’’ stereo jack adapter. Example settings for verifying sound calibration using the MI software are shown in supplementary Fig. S2.

The initial calibration of the mini-microphone was conducted by sound engineers from the Kresge Hearing Research Institute at the University of Michigan. They compared it against a 1/8 inch (3.2 mm) microphone (Brüel & Kjær type 4138; type 2619 preamp, and type 2804 power supply) using a sound source (Krohn-Hite model 4400A Ultra-Low Distortion Oscillator) and a spectrum analyzer (Stanford Research Systems Model SR760). The resulting calibration parameters were then input into the MI audio software according to the developer’s instructions. Following the initial calibration, we routinely verified the calibration parameters using a standard sound level calibrator (1 kHz 94 dB SPL; REED Instruments #R8090).

Commercially available microphones can be utilized in lieu of a custom mini-microphone. We calibrated the acoustic properties of the sounds generated from our sound delivery setup using two additional commercial microphone systems: (1) a 6-mm omni-directional electret condenser USB microphone (Virtins VT RTA-168B) with the Multi-Instrument audio software; (2) a 1/8 inch CCP Pressure Standard Microphone Set (GRAS 46DE) with APx517B amplifier (Audio Precision) and APx500 v6.0 Audio Measurement Software (Audio Precision). These commercial microphones, due to their larger size compared to the custom mini-microphone, may lead to measurement inaccuracies if used for calibrating the sound reaching the worm as described below. Nevertheless, these larger microphones can still be used for calibrating the sound output from the speaker as long as they are kept consistent throughout the experiments.

### Calibration procedure for mimicking worm conditions

To ensure consistency of the sound stimulus reaching the worm, we calibrated the sound reaching the worm using the sound calibration system described above (Fig. 2). We recommend performing sound calibration, if possible, in an anechoic chamber to shield background noise. If this is not available, it is advised that background noise is <50 dB as measured using the mini-microphone. Background noise can be further reduced by insulating the experimental area with sound-dampening panels. We calibrated the speaker settings while mimicking experimental conditions. For behavior and vibration experiments, the horizontal distance between the mini-microphone and speaker opening was fixed by first placing a millimeter-marked scale beneath the stereo microscope’s field of view with a standard nematode growth medium (NGM) plate placed on top, akin to the experimental setup (Fig. 2A). Using the scale as a reference, we gently pressed on the agar surface of the NGM plate at 1-mm intervals with a platinum wire to create shallow marks, marking at least two points (labeled points A and B, see Fig. 2A), before removing the underlying scale. We next adjusted the micromanipulator to lower the tip of the speaker just above one of the marked points, labeled A (Fig. 2A). The speaker tip was then lowered to the agar surface at point A, taking care to not indent or damage the agar, then raised 1 mm above the agar surface (z-direction) using the Vernier caliper equipped on the micromanipulator (Fig. 2A). Next, we positioned the mini-microphone so that its aperture was 1-mm adjacent to the speaker opening in the *x*-direction, positioned at point B, ensuring that the mini microphone's opening is parallel to that of the speaker (Fig. 2A). The microphone’s position was maintained using modeling clay to secure the wire to the microscope base. The sound intensity was then calibrated to determine the input settings that resulted in the desired intensity via the microphone using the MI software. We adapted a similar method to calibrate the speaker for calcium imaging experiments, with the only notable difference being that the horizontal distance between the worm and speaker opening was increased to 4 mm. Calibration was performed before each experiment to ensure precise control over the sound intensity reaching the worm.

### Phonotaxis behavioral assay

The sound-evoked phonotaxis behavior was assessed on Day 1 adult hermaphrodite worms. Worms were tested on NGM plates freshly poured between 1–5 days or stored at 4 °C before use to prevent excessive drying. Prior to the experiment, 50 µL of fresh OP50 bacteria were seeded onto the testing plates and dried immediately with the lid off. Hermaphrodite worms at the L4 stage were picked one day before the experiment and transferred to freshly seeded NGM plates 10 min before testing.

The experimental setup was similar to that used for calibrating speaker outputs. While observing under the stereo microscope, the speaker was kept stationary while moving the NGM plate so that the worms’ body was parallel to the speaker aperture and positioned at a distance equivalent to one body length of the nematode (~1 mm) (Fig. 1). A 2-s pulse of sound at 80 dB SPL (1 kHz) was then delivered to the head of a forward moving worm for each trial. A successful head-avoidance response was scored if the worm stopped forward movement and reversed at least half of one head-swing within 5 s from the onset of the sound stimulus (in our case, since our tone consisted of a 2-s pulse, this was from stimulus onset to within 3 s after the sound stimulus ceased). The recommended sample size is 10 for each experiment. This can be done by seeding 5–10 worms per plate with each worm tested once, and the average percentage score of backward movement of these worms is tabulated as one data point. Alternatively, each plate may house one worm, which is tested five to ten times with a 5–10 min interval between tests to stabilize behavior, scoring the average percentage of backward movement of the worm across all trials as one data point. The latter protocol may capture the adaptation property of phonotaxis responses. To ensure consistent behavioral phenotypes, all experimental conditions were repeated with independent cohorts of worms at least once.

### Laser Doppler vibrometry

**Figure 3:**
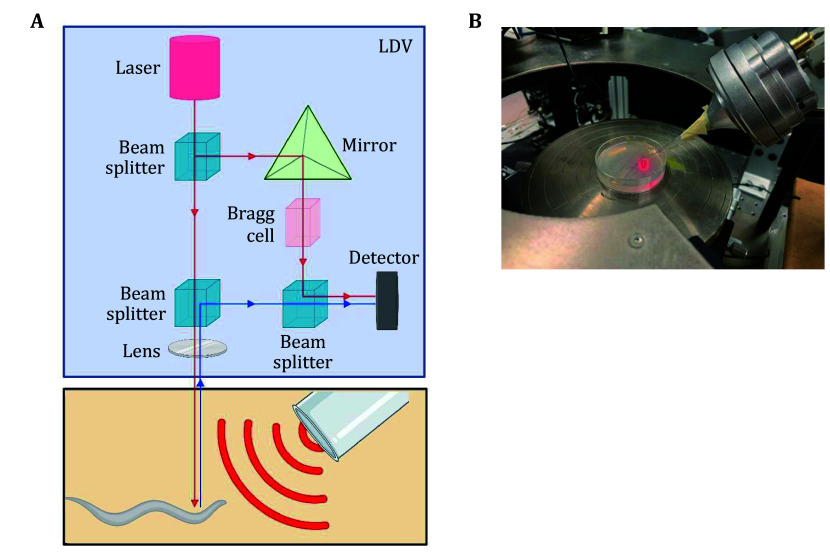
Overview of the laser Doppler vibrometry (LDV) system to measure non-contact surface vibrations. **A** Schematic describing the components of the laser Doppler Vibrometry (LDV) system used to measure surface vibrations. The laser beam was directed at the surface of either the *C. elegans* anterior body or adjacent underlying agar substrate. The Doppler shift of the reflected laser beam enables quantification of surface vibration (displacement) of the worm or agar substrate. **B** Snapshot image of the sound delivery system positioned beneath the LDV system. The sound delivery system is positioned towards the laser spot mimicking calibration and behavior assay conditions. An NGM plate containing paralyzed worms can be positioned beneath the laser spot to measure the surface vibrations of the worm or agar substrate

To measure sound-evoked surface vibrations in both *C. elegans* and the background substrate, we employed a laser Doppler vibrometer (LDV; OFV-303, Polytec USA) to quantify superficial vibrations (Goode *et al*. 1996) (Figs. 3A and 3B). We found that sound administered via our described sound delivery setup induces no or minimal vibration of the substrate surface but robustly vibrates the worm cuticle (Iliff *et al*. 2021). Here we describe a method to measure sound-evoked surface vibrations by laser Doppler vibrometry. To do so, the LDV output was connected to a computer using an NI PCI-6123 card. For vibration measurements at 1 kHz frequency, the acquiring voltage (mm/(s⦁V)) was set for a duration of 1 s at a sampling rate of 10 kHz. A custom MATLAB script (available upon request) was used to post-process voltage data using a set conversion factor of 10 mm/(s·V) to measure displacement in relation to frequency. For all vibration measurements, a focused laser spot size with a diameter of 10 µm was aimed at the target (worm body or NGM agar substrate) (Fig. 3B).

The dryness of NGM plates used for measurements was carefully standardized, as excessive moisture or variability in plate dryness may impact vibration measurements. Thinner volumes of agar (3 mL of NGM poured fresh in 55-mm dishes) were consistently standardized in terms of plate dryness. We poured 3 mL of fresh NGM for use two days prior to the experiment and allowed the plates to dry at room temperature overnight. Plates were either used the next day or wrapped in parafilm and stored at 4 °C for up to one week, allowing for overnight drying at room temperature to remove excess moisture. Prior to the experiment, plate dryness was standardized by measuring the absorption time of a 1 µL M13 droplet into the NGM surface; an absorption time of 3–5 s was deemed optimal. Conditions were confirmed by observing normal phonotaxis behavior.

To measure sound-evoked skin vibrations in worms, plates were also treated with 100 uL of 10 mmol/L NaN3 (in M13 solution) spread as a thin layer along the agar surface and allowed to absorb overnight prior to the experiment, ensuring appropriate dryness (via timing absorption of M13 droplet) prior to vibration measurements. To place worms on the plates for measurements, we first paralyzed them on an unseeded NGM plate in a droplet of 10 mmol/L NaN3 (in M13) until movement ceased. The remaining liquid around the worm was allowed to dry completely using a Kim wipe to wick any excess liquid away if necessary. Worms were transferred using an eyelash pick to the testing plates (10 worms per test), orienting the worms in parallel on the agar surface in the center. The laser was focused on the anterior mid-body of the worm just behind the pharynx. Measurements were taken at baseline (without sound stimulus) and in response to the sound stimulus with the speaker positioned as described for the behavior assay. Young adult worms (Day 1–2) were used for experiments, standardizing the exact age of worms across genotypes for each test. For strains with disrupted cuticle *bli* mutants (Cohen and Sundaram 2020), Day 2 worms were chosen to assay as the *bli* phenotype becomes more penetrant at Day 2. For *bli* mutants, the laser was focused on an area just behind the pharynx with a disrupted cuticle from the anterior blister. Ten individual worms or the adjacent agar surface were tested for each measurement.

### Calcium imaging assay

Calcium imaging was performed in an environmentally controlled room (20 °C, 30% humidity). Prior to each experiment, the sound stimulus (10 s, 1 kHz at 89 dB SPL) from the sound delivery system was calibrated as described above. Worms carried a transgene driving GCaMP6 and mCherry expression in the sound-sensing FLP neurons using the *sto-5* promoter (Russell *et al*. 2014) to enable ratiometric imaging. Here, we describe methods to image both freely moving and immobilized worms to record the calcium activity of FLP neurons in response to auditory stimuli. A similar method can be used to record the other sound-sensitive neuron PVD.

**Figure 4:**
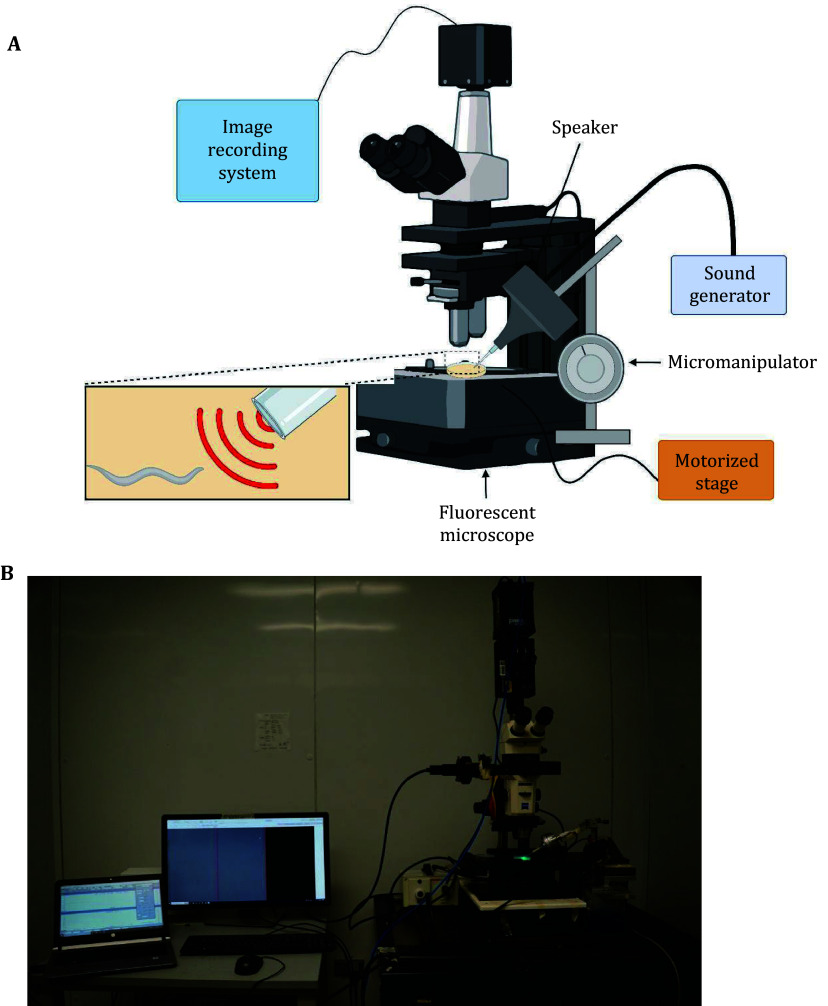
Overview of the method used to record neuronal responses to sound stimulation in freely moving worms. **A** Schematic depicting the integration of the sound delivery system with a previously described custom freely-moving calcium imaging system (CARIBN) (Iliff *et* *al*. 2021; Piggott *et al*. 2011). Prior to imaging, the sound delivery system is calibrated in an identical position to ensure the accuracy of the sound intensity reaching the worm. Sound-evoked neuronal activity of a single neuron is monitored across time in a freely-moving worm. **B** Snapshot image of the CARIBN imaging system in use with the sound delivery system to record sound-evoked neural activity

To record sound-evoked neuronal responses in freely moving animals, we utilized a customized calcium imaging system known as CARIBN (Figs. 4A and 4B) (Piggott *et al*. 2011; Zheng *et al*. 2012). This system is equipped with a motorized stage to track worm movement. Testing plates were prepared following the procedure outlined for phonotaxis behavior. A single Day 1 adult hermaphrodite worm was delicately transferred to a testing plate using an eyelash pick, allowing for a 10-min stabilization period prior to recording. Each worm underwent testing once only to prevent adaptation to sound stimuli. After the 10-min habituation period, the testing plate, with lid removed, was positioned on the automated tracking stage assembled beneath the fluorescence microscope, enabling precise movement tracking using the CARIBN system. Manual adjustments were made to achieve a clear image of a single neuron. It is recommended to manually hold the fine focus knob of the fluorescence microscope to prevent defocusing during image recording. Recording commenced after a 3-min stabilization period to adapt the worms to blue light (Ward *et al*. 2008; Liu *et al*. 2010). Alternatively, one may use *lite-1* light-insensitive mutant worms for imaging (Ward *et al*. 2008; Liu *et al*. 2010; Gong *et al*. 2016). Each recording session required a minimum 60-second time window. A 10-s pre-stimulus recording was collected to quantify baseline fluorescence intensity, followed by a 50-s recording to observe real-time neuronal activity during sound stimulation and recovery.

For recording sound-evoked neuronal responses in immobilized worms, most upright microscopes equipped with an epi-fluorescence system, including stereo and compound microscopes, should suffice, without the need of using a motorized stage. As the sound stimulus is administered from above, upright but not inverted microscopes should be used. We recommend equipping the microscope with a 10x or 20x air objective in conjunction with a mounted sCMOS or EMCCD camera to record fluorescence intensity. An upright confocal microscope could also be utilized if the working distance between the stage and the objective is wide enough, ensuring the tip opening attached to the speaker to position near the worm. In addition, a customized micromanipulator mounted with the speaker would also enable the accessibility of the tip opening to the worm head.

To prepare the immobilized worm for calcium imaging, a single Day 1 adult hermaphrodite worm was first transferred to a suitable substrate (2% agarose pad or testing plate) for imaging. It is important to ensure phonotaxis behavior persists on the substrate used for calcium imaging of auditory responses. Therefore, we recommend using the testing plates as the substrate if possible to minimize potential variations in observed results. Immobilization of worms can be achieved by applying either cyanoacrylate adhesive glue or chemical paralytics (*i*.*e*. levamisole) (Chung *et* *al*. 2013). To precisely administer glue, we recommend using a fine capillary needle prepared from a pulled borosilicate glass capillary (Sutter BF150-86-10). The needle was loaded with 10 uL of cyanoacrylate adhesive glue using a microloader tip (Calibre Scientific EPE-930001007). To precisely control the administration of glue flowing from the needle, we recommend attaching tightly fitting tubing to the back of the needle connected to a syringe to push tiny but visible amounts of glue onto one side of the worm’s body so that movement ceases. Care should be taken to avoid excessive glue, as this may (1) desiccate the worm and (2) interfere with the transduction of auditory stimuli.

Alternatively, worm immobilization can be achieved using 5 mmol/L levamisole (Chung *et al*. 2013) (in standard M9 solution). Day 1 adult hermaphrodite worms were delicately transferred to a testing plate using an eyelash pick, allowing for a ten-minute stabilization period. To achieve immobilization during imaging, a drop of 5 µL of 5 mmol/L levamisole was placed on top of a single worm on a testing plate and allowed to incubate in the droplet for 5 min or until the drop was absorbed into the agar surface. Once worms were immobilized by either method, the same imaging procedure for recording GCaMP/mCherry fluorescence can be performed as described above for freely moving worms. It should be noted that as the FLP neuron is mechanosensitive, it is easily pre-activated by the immobilization procedure, leading to a higher basal fluorescence level. Consequently, calcium imaging performed on immobilized worms may show a smaller ratio change in GCaMP6/mCherry fluorescence compared to that recorded in freely moving worms. We recommend resting the worm for 5 min to stabilize the basal calcium activity of the neuron before imaging.

Ratiometric imaging in freely moving and immobilized worms was conducted on worms co-expressing GCaMP6(f) and mCherry, and Δ*R*/*R* was used to quantify changes in fluorescence. The baseline fluorescence level was recorded for at least 10 s prior to sound stimulation. We quantified the peak calcium response, as it was found to be more consistent between trials.

## RESULTS

To examine *C. elegans* auditory sensation, we first constructed a sound delivery system capable of administering precise, localized pure tones with frequencies ranging from 100 Hz to 5 kHz and a maximal intensity of 110 dB SPL (Figs. 1A–1C). While this method can be adapted according to individual user needs, it is important to consider certain essential parameters outlined here when designing a sound delivery device in order to optimize *C. elegans* auditory responses. For example, as worms sense sound pressure gradients rather than absolute sound pressure levels, they selectively respond to localized sounds (Wang *et al*. 2023). We thus recommend designing a system with a small speaker size (*e*.*g*., 1.5-mm inner diameter or smaller). The described speaker opening can be readily modified by cutting the attached pipette tip to various sizes as appropriate.

It is also essential to carefully calibrate the sound delivery device to ensure that precise pure tones are generated prior to use. To facilitate this, we have provided a detailed method for sound calibration aimed at ensuring the accuracy of delivered sounds, which can be utilized irrespective of the chosen setup design (Fig. 2A). To reduce variability in sound calibration results, we recommend mimicking testing conditions during the calibration procedure. We recommend calibrating the intensity for delivered sounds after placing the microphone on top of the surface of the NGM agar substrate. This approach ensures the delivery of accurate localized sound to worms, thereby eliciting the most robust auditory response. Lastly, as background noise may affect auditory sensation, we recommend performing experiments in an environment with low background noise or, if possible, in an anechoic chamber.

**Figure 5:**
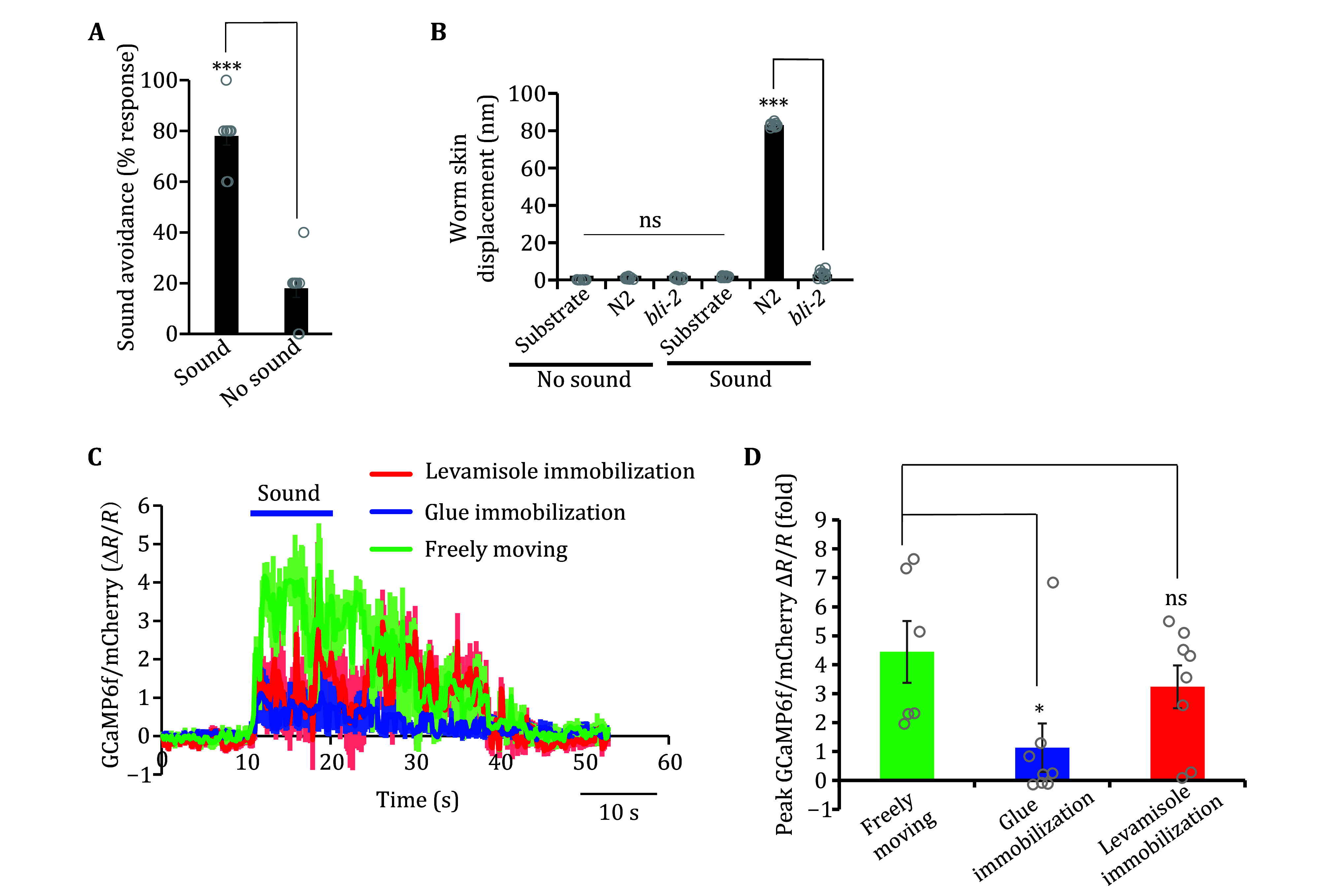
Quantification of *C. elegans* auditory responses using the sound delivery system. **A** Bar graph depicting the head-avoidance phonotaxis response to sound (2 s, 1 kHz, 80 dB SPL). The bar shows the mean value of the plotted individual data points. ∗∗∗*p* < 0.0001 (*t-*test). *n* = 10 for each condition. The low basal response observed in the no-sound group was due to baseline spontaneous reversals. **B** Bar graph depicting the displacement values of surface vibrations measured using the laser Doppler vibrometry (LDV) system. Measurements of the agar substrate or worm body surface were taken at baseline or evoked by sound (1 kHz, 80 dB SPL). Cuticle-defective *bli* mutants show a strong defect in sound-evoked vibrations compared to N2 (wild-type) worms. The bar shows the mean value of the plotted individual data points. ∗∗∗*p* < 0.0001 (ANOVA with Tukey test). *n* = 10 for all conditions. **C**,**D** Sound-evoked FLP calcium responses in freely-moving versus immobilized worms. Imaging was performed on worms that carried a transgene co-expressing GCaMP6f/mCherry in FLP neurons using the *sto-5* promoter. Immobilization was performed by either gluing or treatment with 5 mmol/L levamisole. Average traces of calcium responses in FLP neurons. Shades along the traces indicate error bars (SEM) (**C**). Graph depicting the mean values of the peak calcium response in FLP neurons (**D**). The bar shows the mean value. *p < 0.05 (ANOVA with Tukey test). Sound stimulus: 10 s, 1 kHz at 89 dB SPL. *n* ≥ 8 for all conditions

Once the sound delivery system is set up and calibrated, this system can be used to characterize sound-evoked auditory responses. Sound stimuli evoke backward (reversal) and forward movement when delivered to the head and posterior body of the worm, respectively (Iliff *et al*. 2021; Wang *et al*. 2023). As it is easier to assay reversals, here we focused on describing sound-evoked head avoidance response. Our results show that localized sound (1 kHz 80 dB SPL) directed at the head of a forward-moving worm caused it to halt forward movement followed by reversal (Fig. 5A), in line with our previous study (Iliff *et al*. 2021). The low level of “response” observed in no-sound control resulted from spontaneous reversals during locomotion (Fig. 5A). Thus, localized sound can evoke a robust phonotaxis response in *C. elegans*.

We previously discovered that localized sound vibrates the surface of *C. elegans* skin, providing direct evidence that worms respond directly to airborne sound (Iliff *et al*. 2021). Here, we detailed the method employing a laser Doppler vibrometer that enables vibration quantification by measuring surface displacement (Figs. 3A–3E). As sound stimuli have the potential to induce vibrations of any target surface, it is critical to determine whether *C. elegans* directly responds to airborne sound versus secondary mechanical perturbations of the substrate. We thus quantified the displacement of both the agar surface and the worm body under both sound and no-sound conditions. Our results demonstrate that our applied sound stimulus (1 kHz, 80 dB SPL) did not significantly vibrate the agar substrate surface compared to baseline measurements, but did induce significant vibrations compared to its baseline when aimed at the worm’s skin (Fig. 5B), consistent with our previous reports (Iliff *et al*. 2021; Wang *et al*. 2023). Importantly, as reported previously (Iliff *et al*. 2021), the sound-evoked vibrations were greatly diminished in the cuticle-defective *bli-2* mutant worms (Fig. 5B). We do not deem measurements of sound-evoked surface vibrations a requirement to study auditory sensation in *C. elegans* as the LDV equipment may not be commonly accessible. However, to minimize substrate vibrations that may confound studies of worm auditory sensation due to potential secondary activation of other mechanosensory pathways, we recommend closely following our instructions in setting up the sound delivery system. If the system is vastly modified, we recommend the described method to measure the superficial vibrations of *C. elegans* and their substrate.

The multidendritic mechanosensory neurons FLP and PVD are primary auditory receptor neurons that sense localized sounds to elicit backward and forward movement, respectively (Iliff *et al*. 2021). Here, we detailed methods to directly record the activity of these neurons in response to sound stimuli via calcium imaging using the custom sound delivery setup in tandem with calcium imaging of worms co-expressing the genetically encoded fluorescence calcium indicator GCaMP6f and mCherry. Specifically, sound aimed at the worm head (1 kHz 89 dB SPL) of freely moving animals expressing GCaMP6f/mCherry in FLP neurons elicited a robust calcium response in wild-type worms (Figs. 5C and 5D). Here, we also described methods to measure sound-evoked calcium responses in immobilized worms using either glue or levamisole. Our results show that the sound-sensitive FLP neuron in immobilized worms was activated by 1 kHz sound, but the amplitude of the response was not as robust as that in freely moving worms (Figs. 5C and 5D). Furthermore, when worms were immobilized, they demonstrated a significant increase in the FLP basal calcium intensity compared to freely moving worms (supplementary Figs. S3A and S3B). Taken together, this data indicates that while immobilization indeed increases the basal calcium activity in FLP, sound-evoked activation can still be observed, though not as robustly as in freely moving worms. Therefore, imaging immobilized worms provides a convenient alternative method to assay sound-evoked activation of FLP/PVD neurons when the equipment capable of imaging freely moving worms is not available.

**Figure 6:**
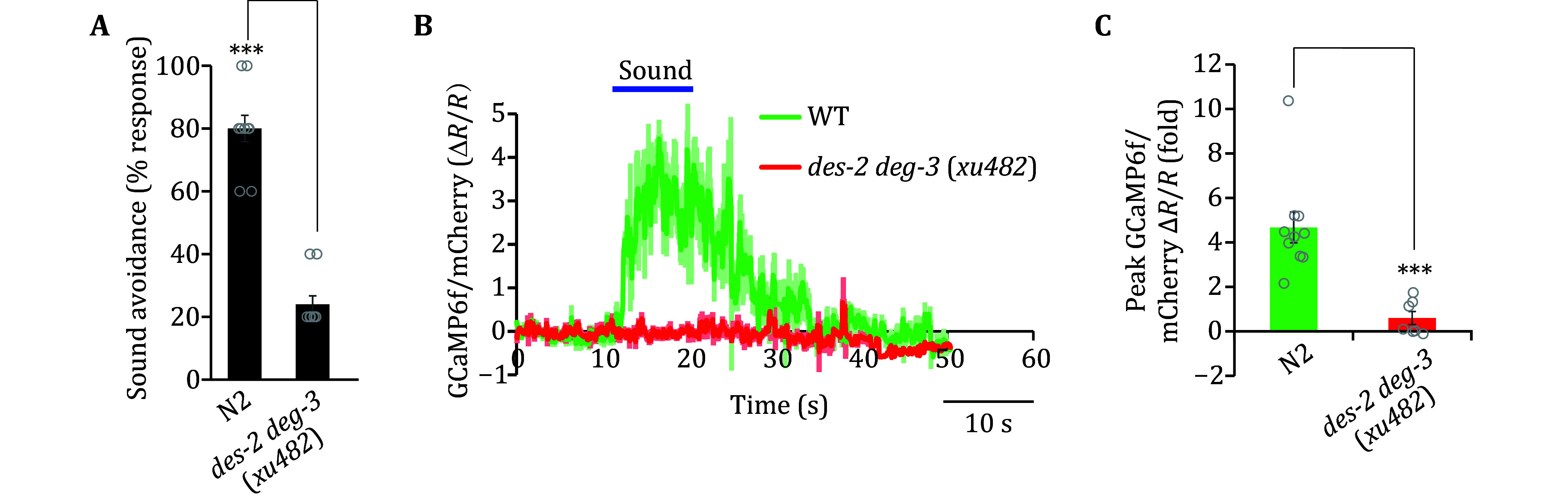
Characterization of mutants defective in auditory sensation using auditory assays. **A** *des-2 deg-3* mutant worms are defective in head-avoidance phonotaxis. The bar shows the mean value of the plotted individual data points. ∗∗∗*p* < 0.0001 (*t*-test). *n* = 10 for each condition. Sound stimulus: 2 s, 1 kHz at 80 dB SPL. N2: wild-type. **B**,**C** Sound-evoked FLP calcium responses show that FLP neuron in *des-2 deg-3* mutant worms fails to respond to sound. **B** Average traces of calcium responses in FLP neurons. Shades along the traces indicate error bars (SEM). **C** Graph depicting the mean values of the peak calcium response from traces shown in Panel B. Bar shows the mean value. Sound stimulus: 10 s, 1 kHz at 89 dB SPL. ∗∗∗*p* < 0.0001 (*t-*test). *n* ≥ 8 for all conditions

Our previous study identified a key role for the nAChR DES-2/DEG-3 in transducing sound stimuli in sound-sensitive FLP/PVD neurons (Iliff *et al*. 2021)*.* Here we also utilized both the behavioral assay and calcium imaging assay to illustrate the necessity of DES-2/DEG-3 in auditory sensation (Figs. 6A–6C). As expected, wild-type worms exhibited robust avoidance of sound (1 kHz 80 dB SPL) while *des-2 deg-3*(*xu482*), the genetically engineered deletion allele, exhibited a pronounced deficiency in phonotaxis response (Fig. 6A). Furthermore, FLP neurons in *des-2 deg-3*(*xu482*) mutant worms failed to respond to sound in the calcium imaging assay (Figs. 6B and 6C). In conclusion, our experiments demonstrate that the outlined methods can be used to characterize three features of auditory sensation in *C. elegans.*

## DISCUSSION

Here, we describe stepwise methods for characterizing auditory sensation in *C. elegans*. First, we present the steps to build a custom sound delivery system for assessing *C. elegans* auditory sensation. The described methods provide the necessary framework and specifications to develop a custom sound delivery device. Custom-designed systems offer the advantages of flexibility in design and cost-efficiency. However, it is imperative to review the specifications for each component to prevent equipment damage or inaccuracies in sound generation. This is especially critical when configuring user-defined parameters, such as the frequency and intensity of the generated tone. For example, setting these parameters outside the recommended ranges according to the manufacturer could irreversibly damage the speaker. In addition, we have recommended several alternative commercial sound generation and calibration systems. This may require less initial troubleshooting during setup but is comparatively less cost-efficient.

*C. elegans* exhibits phonotaxis behavior to sounds directed at the head or tail, resulting in backward or forward locomotion, respectively (Iliff *et al*. 2021). As backward locomotion (reversals) can be more readily scored, we recommend beginning with the head avoidance assay when learning to examine phonotaxis behavior. To examine tail-avoidance phonotaxis, the same experimental conditions described here can be applied while aiming the speaker aperture towards the tail of the worm. Importantly, our prior work indicates that the head of the worm tends to be much more sensitive to sound stimuli than the tail during behavior tests. Therefore, care must be taken when positioning the speaker aperture to avoid directing sound towards the head of the worm, as this will override the tail-avoidance response and instead stimulate a reversal response. As it can also be challenging to accurately detect increases in forward locomotion, for tail-avoidance assay we recommend limiting testing only to worms that are slowly moving forward or non-moving to ensure reliable detection of speed increase.

In general, the administered tones from our described sound delivery device generate minimal mechanical vibrations of the background agar substrate. However, if the system is substantially modified, for example, by using a much more powerful speaker, substrate vibration may become a concern. This may result in the activation of other mechanosensory transduction mechanisms such as the touch receptor neurons (ALM, AVM, PLM, and PVM) (Goodman and Sengupta 2019), which may confound data interpretation. In this case, we recommend confirming by laser Doppler vibrometry that generated tones are not producing significant background vibrations prior to examining features of *C. elegans* auditory sensation. One may also consider testing touch-insensitive *mec-4* mutants that respond normally to airborne sound but are insensitive to substrate-borne vibrations (Iliff *et al*. 2021). If *mec-4* mutants exhibit a deficit in phonotaxis responses compared to wild-type worms, it would suggest a contribution by substrate-borne vibrations.

To examine neuronal activation in response to auditory stimuli, we described two approaches to monitor calcium responses in the sound-sensitive neuron FLP. The same methods can be used to image PVD neuron responses to sound stimulation. Importantly, FLP/PVD neurons exhibit increased baseline calcium levels when animal movement is restricted, which may reduce the dynamic range of the sound-induced neuronal responses. Therefore, if possible, we recommend using a calcium imaging system to record FLP/PVD neurons in freely moving worms when examining auditory sensation. If this is not readily available, worms can be immobilized using levamisole or glue.

## CONCLUSION

Among the six common sensory modalities, hearing is unique in that it had long been thought to be found exclusively in vertebrates and certain arthropods (Budelmann 1992; Faure *et al*. 2009; Webster 1992). This has led to the notion that non-arthropod invertebrates are insensitive to sound (Budelmann 1992; Faure *et al*. 2009; Webster 1992). Yet, given the evolutionary advantage of hearing for predator and prey detection, the ability to perceive sound may have evolved more widely across the animal kingdom (Iliff *et al*. 2021; Wang *et al*. 2023). Our discovery that *C. elegans* senses airborne sound, which may enable these animals to detect and avoid predators, lends support to this notion. This establishes *C. elegans* as an excellent model organism for the study of auditory sensation. Much remains to be discovered regarding auditory sensation in *C. elegans*. The methods detailed here provide an accessible platform to characterize auditory sensation and mechanotransduction in *C. elegans* and reveal the underlying molecular and neural mechanisms. We anticipate that this will be useful for a spectrum of applications ranging from teaching environments to research laboratories.

## Conflict of interest

Can Wang, Elizabeth A. Ronan, Adam J. Iliff, Rawan Al-Ebidi, Panagiota Kitsopoulos, Karl Grosh, Jianfeng Liu and X.Z. Shawn Xu declare that they have no conflict of interest.
